# Anticolorectal Cancer Effects of AUCAN: Effects to Suppress Proliferation, Metastasis, and Invasion of Tumor Cells

**DOI:** 10.1155/2020/9786428

**Published:** 2020-10-10

**Authors:** Liu-Lin Xiong, Ruo-Lan Du, Jun-Jie Chen, Ya Jiang, Lu-Lu Xue, Rui-Ze Niu, Li Chen, Jia Liu, Ting-Hua Wang

**Affiliations:** ^1^Institute of Neurological Disease, West China Hospital, Sichuan University, Chengdu 610041, Sichuan, China; ^2^Department of Anesthesiology, Affiliated Traditional Chinese Medicine Hospital, Southwest Medical University, Luzhou 646000, Sichuan, China; ^3^Institute of Neuroscience, Animal Zoology Department, Kunming Medical University, Kunming 650031, Yunnan, China

## Abstract

**Background:**

Colorectal cancer (CRC) is an underlying deadly malignancy with poor prognosis, lacking effective therapies currently available to improve the prognosis. C_18_H_17_NO_6_ (AUCAN), a kind of dibenzofuran extracted from a special plant in Yunnan Province (China), is identified as a natural anticancer agent exerting strong inhibitory activities on various cancers. Our study was committed to investigating the potency of AUCAN against colorectal cancers and further exploring the potential mechanisms via proteomic analysis.

**Methods:**

Cell Counting Kit-8 assay and immunofluorescence staining were used to investigate the effect of AUCAN on the viability and proliferation of HCT-116 cells and RKO cells. The apoptosis of HCT-116 and RKO cells after AUCAN administration was determined by the flow cytometry test. The effects of AUCAN on invasion and migration of tumor cells were investigated by the colony formation assay, wound healing test, and Transwell invasion test. Meanwhile, the energy metabolism and growth of tumor tissues after AUCAN administration with 10 mg/kg and 20 mg/kg were examined by PET-CT *in vivo*. The side effects of AUCAN treatment were also evaluated through blood routine and liver function examination. RKO cell proliferation and apoptosis *in vivo* were further determined by hematoxylin and eosin staining, TUNEL staining, and immunohistochemistry. Furthermore, the differentially expressed proteins (DEPs) involved in AUCAN treatment were determined by proteomic analysis followed by functional clustering analysis.

**Results:**

The results showed that AUCAN suppressed the migratory abilities and enhanced apoptosis of HCT-116 and RKO cell lines. Meanwhile, AUCAN treatment dramatically depressed the growth and volume of colorectal tumors in nude mice and suppressed the survival of RKO cells in tumor tissues without any side effects on the blood routine and liver function. In addition, twenty-four upregulated and forty-two downregulated proteins were identified. Additionally, functional clustering analysis concealed enriched biological processes, cellular components, molecular functions, and related pathways of these proteins involved in cellular metabolic. Finally, the protein-protein interaction analysis revealed the regulatory connection among these DEPs.

**Conclusions:**

Taken together, AUCAN exerted its significant antitumor effect without side effects in the blood routine and liver function and the underlying mechanisms were preliminarily investigated by proteomic analysis.

## 1. Background

Colorectal cancer (CRC), also called as bowel cancer and colon cancer, represents the third most common cancer among males and the second most common cancer among females worldwide [1]. In developed countries, the onset age is over 50 for more than 90% of patients, but in developing countries, the diseased population is younger [2]. A number of individual factors, including first-class family history (FHCRC) and inflammatory bowel disease [3, 4], are related to the increased risk of CRC. The patient's health, preferences, and tumor grade [5] decide that colorectal cancer is treated in a variety of ways, including laparotomy and chemotherapy, radiotherapy, immunotherapy, and palliative care [2, 6, 7]. Clinically, though these therapies are curative, various side effects still exist. It is therefore utmost essential to determine diagnostic biomarkers which contribute to further identify potential mechanisms for the treatment of CRC.

The application of traditional Chinese medicine (TCM) in cancer treatment has a long history. Patients mainly benefit from traditional Chinese medicine in immune regulation, efficacy improvement, adverse reactions reduction, and drug resistance elimination [8, 9]. C_18_H_17_NO_6_ (AUCAN), known as a dibenzofuran extracted from a special plant in Yunnan Province (China), has been identified as a natural anticancer agent exhibiting strong inhibitory effect on a large number of cancers with low toxicity (patent ID: 201710388136.8). What is more, the purity of the compound reaches 99.5%. AUCAN had been reported to explore in breast cancer, liver cancer, lung cancer, bladder cancer, and nasopharyngeal carcinoma *in vitro* [10], the antitumor effect which is achieved by affecting cell metabolism, proliferation, and cell cycle distribution [10]. However, AUCAN has been seldomly reported to be associated with CRC and little is known about the underlying mechanism of AUCAN in CRC.

Here, we explored the antitumor efficacy of AUCAN in CRC by applying human-sourced HCT-116 and RKO colon cancer cell lines as well as CRC mice. Our findings demonstrated the suppressive activities of AUCAN on the growth, angiogenesis, and metastasis of colorectal cancer cells *in vitro* and *in vivo* and evidently revealed its potential mechanism via proteomic analysis.

## 2. Materials and Methods

### 2.1. Cell Culture

Colorectal carcinoma cell lines HCT-116 (ATCC number: CCL-247) and RKO (ATCC number: CRL-2577), purchased from Kunming Institution of Zoology, were cultured as previously described [11]. HCT-116 cells were grown in RPMI medium modified (Hyclone, USA) with 10% fetal bovine serum (FBS; Hyclone, USA) and 1% penicillin-streptomycin solution (PSS, Hyclone, USA). RKO cells were cultured in DMEM/high glucose (Hyclone, USA) medium containing 10% FBS and 1% PSS. After 2 washes with phosphate-buffered saline (PBS; Hyclone, USA), the cells were digested for 3 minutes (min) with 0.25% trypsin (Gibco) and later was ended by FBS-containing medium. Afterwards, cells were centrifuged at 800~1000 rpm for 5-8 min, the cell suspension was obtained, and the cells were plated in 25 T (3 ml) culture flasks at a density of 4 × 10^5^ cells/ml in an incubator. After being incubated for 24 hours (h), the supernatant was replaced with the fresh medium. When they reached 90% confluency, the medium was changed every 3-5 days (d) and the cells were subcultured. The pure adherent HCT-116 and RKO cells were chosen for the later experiments. The growth status of the cells was observed under an inverted microscope (Leica, Germany).

### 2.2. AUCAN (C_17_H_17_NO_6_) Administration *In Vitro*

To screen out the best effective concentration of AUCAN administration in colorectal tumor cells, AUCAN (LongJing Biotech, Kunming, Yunnan, China) concentration in Cell Counting Kit-8 assay was set as 1 *μ*M, 3 *μ*M 5 *μ*M, 7 *μ*M, 9 *μ*M, 11 *μ*M, 13 *μ*M, 15 *μ*M, and 17 *μ*M for the HCT-116 cells, as well as 0.3 *μ*M, 0.5 *μ*M, 1 *μ*M, 3 *μ*M, 4 *μ*M, 5 *μ*M, 7 *μ*M, 9 *μ*M, 11 *μ*M, and 13 *μ*M for the RKO cells, and administered into the previously cultured HCT-116 and RKO cells (*n* = 5 wells per group). For the following *in vitro* and *in vivo* experiments, the drug doses for HCT-116 cells were set as 8 *μ*M (AUCAN-IC50) and 3.2 *μ*M (AUCAN-IC20), as well as 5 *μ*M (AUCAN-IC50) and 2 *μ*M (AUCAN-IC20) for RKO cells. The control groups were added with 8 *μ*M DMSO (HCT-116 cells) and 5 *μ*M DMSO (RKO cells), respectively.

### 2.3. Cell Counting Kit-8 (CCK-8) Assay

The viability of HCT-116 and RKO cells after AUCAN administration was detected using CCK-8 assays. HCT-116 and RKO cells were plated into 96-well plates at a density of 3000 cells per well in medium containing 10% FBS (Hyclone, USA) and 1% PSS (Hyclone, USA), followed by AUCAN treatment and incubation overnight (*n* = 5 wells per group). After coincubation of cells with AUCAN addition, cell viability was valued by CCK-8 assay. Cells in each well were administered with CCK-8 solution (10 *μ*l per well), and 4 h later, the cell viability was determined by measured optical density (OD) at 450 nm under Thermo Scientific Microplate Reader (Multiskan GO, Thermo). All the procedures were arranged in triplicate and carried out repeatedly three times. The inhibition ratio of AUCAN was expressed as a percentage of the control treated with vehicle solutions.

### 2.4. 5-Ethynyl-2′-Deoxyuridine (EDU)

EDU (RIB BIO) staining was used to access the proliferation of HCT-116 and RKO cells after AUCAN administration. Before EDU solution addition, resuspension and culture of HCT-116 and RKO cells as well as AUCAN administration were performed as described above (*n* = 5 *w*ells per group). EDU solution was then dissolved in medium (1 : 1000) to prepare 50 *μ*M EDU medium, and cells in per well were successively incubated with 50 *μ*M (100 *μ*l/well) EDU medium for 2 h. Next, cells were rinsed with PBS for 2 times (5 min each time). Afterwards, cells were fixed in 100 *μ*l PBS containing 4% paraformaldehyde for 30 min and incubated with 2 mg/ml glycine in rocker for 5 min as well as incubated with 100 *μ*l PBS containing 0.5% Triton 110 in rocker for 10 min. Cells were washed with PBS between each two steps. Next, cells were reacted in 100 *μ*l 1XApollo® in rocker (dark at room temperature) for 30 min and washed for three times in 100 *μ*l 0.5% PBS containing Triton 110 (10 min each time). Addition of 100 *μ*l methyl alcohol and PBS was then added into per well for washing for once (5 min each time), respectively. Lastly, 1× Hoechst 33342 reaction solution was prepared in dark by diluting reagent F with deionized water (1 : 100) and cells per well were incubated with 100 *μ*l 1× Hoechst 33342 for 30 min. Then, 100 *μ*l PBS was added into each well for washing for three times, followed by the addition of 100 *μ*l PBS into each well for further usage. Fluorescence observation and image collection were conducted immediately via confocal microscope (Cell Insight CX5, Thermo, USA).

### 2.5. Flow Cytometry Analysis

The cell cycle and apoptosis of HCT-116 and RKO cells were observed by flow cytometry using a cell apoptosis analysis kit (APC Annexin V Apoptosis Detection Kit with PI) as described before [11]. In short, after AUCAN administration, digestion of cells was followed with 0.25% EDTA-free trypsin, which was then centrifuged for 10 min at 1000 rpm (*n* = 5 wells per group). Cells were collected and resuspended once with 1× PBS (4°C), and centrifuged again for 10 min at 1000 rpm. Cells were then fixed and stained with propidium iodide (PI) for cell cycle assay, while for cell apoptosis assay, cells were resuspended with addition of 500 *μ*l 1× binding buffer containing 4-(2-hydroxyethyl)-1-piperazineethanesulfonic acid, 140 mmol/l NaCl, and 5 mmol/l CaCl_2_ at pH 7.4, and then 5 *μ*l Annexin V-FITC and 5 *μ*l PI were added into the 100 *μ*l cell suspension, followed by incubation in dark at room temperature for 15 min. FACSVerse laser flow cytometry analysis system (Becton Dickinson, USA) was used for analysis.

### 2.6. Colony Formation Test

The cells in logarithmic growth period were digested with trypsin and washed twice with PBS (Hyclone, USA). The number of cells was counted at room temperature, and the cells were prepared into suspension (*n* = 5 wells per group) which was then plated into 6-well plates at a density of 1000 cells per well. After incubation of the inoculated cells under normal conditions at 37°C for 24 h, AUCAN at certain concentration was administrated. Cells were incubated for 8-14 d till the number exceeded 50 in individual well. In addition, the medium was replaced every 3 d and the status of cells was observed. After washes with PBS, cells were fixed in 600 *μ*l of 4% paraformaldehyde at room temperature for 30 min and stained with 200 *μ*l 0.5% crystal violet at room temperature for 10-20 min. Following extensive washes with ddH_2_O for several times, the cells were observed under digital camera for colony counting.

### 2.7. Wound Healing Test

The migration status of HCT-116 and RKO cells after AUCAN administration was determined using the wound healing test as previously described [11]. Firstly, cells were counted at room temperature and cell suspension was prepared (*n* = 5 wells per group) which was then plated into 6-well plates at a density of 5 × 10^5^ cells/well. Subsequently, a plastic 10 *μ*l pipette tip was utilized to make equal-width linear scratches on the 6-well plates. Cell fragments were washed out of scratches with PBS. The images 0 h, 12 h, and 24 h after AUCAN treatment were obtained under multifunctional automated inverted fluorescence microscopy (Nikon). The migration rate is calculated as (the average distance at 0 h − the average distance at x h)/(the average distance at 0 h) × 100%.

### 2.8. Transwell Invasion Assay

The invasion and migration abilities of HCT-116 and RKO cells after AUCAN administration were detected by Transwell invasion assay as described [11]. Briefly, Transwell was put in a new 24-well plate with 100 *μ*l of serum-free medium added to the upper chamber which was then incubated at 37°C for 1 h. Subsequent suspension in serum-free medium was undergone with the specified concentration of drug at a seeding density of 1 × 10^5^/well (*n* = 5 wells per group). In addition to 100 *μ*l of cell suspension to the upper chamber, the lower chamber received 600 *μ*l of 30% FBS medium. For the invasion test, 500 *μ*l of serum-free medium was added to both the upper and lower chambers that were incubated at 37°C for 2 h. Apart from 500 *μ*l of cell suspension to the upper chamber, the lower chamber received 750 *μ*l of 30% FBS medium. Incubation was again continued at 37°C. Forty-eight hours later, nonmigrating and noninvading cells in the chamber were cleaned out with a cotton swab, while migrating and invading cells were fixed with 4% paraformaldehyde for 20 min. After washes with PBS, cells were stained with 600 *μ*l of 0.1% crystal violet at room temperature for 20 min. Five fields of each membrane were captured randomly using a fluorescence microscope (DM4000 B, Leica). The number of migrating or invading cells was calculated as the average number of cells per microscopic field over five fields.

### 2.9. Animals and Groups

Thirty female nude mice aged 4 weeks (weighing around 18-22 g) were provided by Kunming Medical University and housed in a specific pathogen-free (SPF) environment. They were randomly arranged into 3 groups: control, AUCAN 10 mg/kg, and AUCAN 20 mg/kg groups (*n* = 10 per group). Mice in the negative control group were treated with equivalent DMSO, and RKO cell line-transplanted animals in the other 2 groups were administered with 10 mg/kg AUCAN and 20 mg/kg AUCAN, respectively. All experimental protocols were approved by the Animal Care and Use Committee of Kunming Medical University in Yunnan Province, China. Animal handling procedures conformed to the National Institutes of Health *Guide for the Care and Use of Laboratory Animals*.

### 2.10. RKO Cell Transplantation and AUCAN Administration in Nude Mice *In Vivo*

200 *μ*l of 2*E* + 7/ml RKO cells was subcutaneously transplanted into the armpit of the right forelimb of each nude mouse. Then, the tumor growth status was observed after injection for 5-7 d in the control, 10 mg/kg, and 20 mg/kg AUCAN groups, with 10 mice in each group. Afterwards, rats in the control group were treated with equivalent DMSO, and animals in two groups with drug treatment were intraperitoneally administered with 10 mg/kg and 20 mg/kg AUCAN. Additionally, the mice were treated with AUCAN every other day for 21 d totally. The tumor size was measured by a digital caliper every 3 d, and the volume was calculated as *V* = *π*/6 × (length) × (width)^2^.

### 2.11. Positron Emission Tomography-Computed Tomography (PET-CT)

The glucose uptake of tumor tissues in nude mice can be revealed by PET-CT as described [11]. In detail, 28 d after RKO cell transplantation, rats were anesthetized with intraperitoneal injection of 0.7% pentobarbital sodium (10 *μ*l/g) and fixed in the supine position on PET/CT scanning table (Discovery 690/Elite, GE, USA). The standard protocol of AW Volume Share 5 software underwent for 20 min; the whole-body CT and PET data were obtained. The scanning parameters were set as follows: 120 kV voltage, 260 *μ*A electricity, 0.561 screw pitch, 0.5 s/cycle rotational speed, 3.75 mm thickness and interval, 512 × 512 for CT matrix, and fov = 50 cm × 50 cm. Further, CT and PET images were transferred to AW Volume Share 5 workstation to acquire coronal, sagittal, cross-sectional, and 3D images, together with fusion images of CT and PET images. Eventually, the PET/CT pictures were analyzed with a double-blind approach by two PET/CT reporters. The average value of SUV_max_ was determined through drawing the ROI of tumor.

### 2.12. Tissue Harvest

The animals were sacrificed by cervical dislocation after intraperitoneal injection of 2% pentobarbital sodium (10 *μ*l/g). Afterwards, the tumor tissues were removed and stored at -80°C for immunohistochemistry staining (*n* = 10/group), HE staining (*n* = 10/group), and TUNEL staining (*n* = 10/group), and the blood was collected swiftly for the following analysis including aspartate aminotransferase (AST), alanine transaminase (ALT), white blood cell (WBC), platelet (PLT), and concentration of hemoglobin (HGB). Moreover, the tumor images were captured on the whiteboard and Vernier caliper was also used as a reference to read specific dimensions of tumors.

### 2.13. Hematoxylin and Eosin (HE) Staining

To investigate the morphological changes of tumors, the HE staining was performed. After 28 d of RKO cells and drug injection, all of the mice were sacrificed as described above, and the harvested tumor tissues were extracted. The extracted tumor tissues were then fixed with 4% paraformaldehyde (pH 7.4) in 0.1 ice-cold phosphate buffer at 4°C for at least 72 h. Subsequently, the fixed tumor tissues were embedded in paraffin and sectioned at a thickness of 5 *μ*m; after transferring to the slides, the sections were stained with H&E (C0105; Beyotime Institute of Biotechnology). Afterwards, the morphological changes of the tumor tissues in each group were observed by a light microscope (CX40, Shunyu, Ningbo, China).

### 2.14. Immunohistochemistry (IHC)

In order to test the cellular growth status of Ki67 under two different doses of drug administration, IHC was employed. The tumor sections were obtained from nude mice as described above. Before the experiment was performed, the endogenous peroxidase of the sections should be blocked by washes with 0.01 mol/l PBS then treated with a 0.3% hydrogen peroxide in 20% methanol solution at room temperature for 30 min. During the process, three washes were performed with 0.01 mol/l PBS between every two consecutive steps. To block nonspecific immunostaining, 5% goat serum in 0.01 mol/l PBS was used to treat the sections for 1 h at room temperature. Subsequently, the sections were incubated with Ki67 (1 : 1000; Kit-0005, LabVision) in 0.1% Triton X-100/0.01 mol/l PBS overnight at 4°C. After washed in 0.01 mol/l PBS for three times, the sections were incubated overnight with horseradish peroxide-labeled secondary antibody (mouse, 1 : 200, Danish Dakor) in 0.01 mol/l PBS at 4°C. The bound antibodies were visualized with 3,3-diaminobiphenyl tetrahydrochloride (DAKO) by the avidin-biotin-peroxidase complex method in accordance with the standard protocol (Vector Laboratory, Burlingame, California, USA). To test the specificity of the reaction, the negative control slide was treated in the same way as the original antibody was omitted and run with other samples. The images were obtained by an Axiovision 4.6 software system (Karl Zeiss, Germany). Five fields of each slice were randomly collected at 200x, and the positive Ki67 cells (dark brown labeled) in each field were quantified by Image-Pro Plus 6.0 software (Media Cybernetics, Silver Spring, MD, USA).

### 2.15. Terminal Deoxynucleotidyl Transferase-Mediated Nick End Labeling (TUNEL) Staining

The apoptosis of RKO cells in nude mice before or after AUCAN administration was detected by TUNEL staining as described [11]. Briefly, after washes for three times with PBS, sections were firstly fixed with 4% paraformaldehyde for 10 min. With subsequent washes in PBS again, sections were placed in a 37°C incubator with a mixture of 0.1% sodium citrate and 0.3% Triton X-100 for 30 min. The TUNEL reaction mixture was prepared in dark: enzyme solution and label solution at a ratio of 1 : 9 on ice. The sections were then incubated in a dark box at 37°C for 1 h, and DAPI containing antifluorescence quencher was added to stain the cells which were subsequently incubated for 3 min at room temperature. Five fields were randomly chosen out of each slice for imaging at 200x with a fluorescence microscope (Leica, CM1860, Germany). Then, quantification was carried out by Image-Pro Plus 6.0 software. Apoptotic cells (%) were presented as (TUNEL^+^ cell amounts/total cell amounts)%.

### 2.16. Proteomic Analysis

The proteome changes were determined by proteomic analysis in this study. After AUCAN administration for 48 h, RKO cells were collected with 6 × 10^6^ cells for each sample in the AUCAN group (6 × 10^7^ cells for each in the control group). As previously described [12], protein extraction, digestion, peptide labeling, peptide fractionation, phosphorylated peptide enrichment, and mass spectrometer (MS) detection were conducted at Shanghai Luming Biotechnology Co., Ltd. (Shanghai, China).

### 2.17. Statistical Analysis

One-way analysis of variance (ANOVA) with Tukey's post hoc test was applied for multiple group comparisons. And the data between two groups were analyzed using Student's *t*-test. In addition, nonlinear regression analysis was used to analyze IC50 of AUCAN treatment in RKO and HCT-116 cells. Data were presented as the mean ± SD. All statistical analysis was performed using SPSS 13.0 software (SPSS, Inc., Chicago, IL, USA). Statistical significant difference was determined when *P* < 0.05.

## 3. Results

### 3.1. IC50 of AUCAN in HCT-116 and RKO Cells

**Figure 1:**
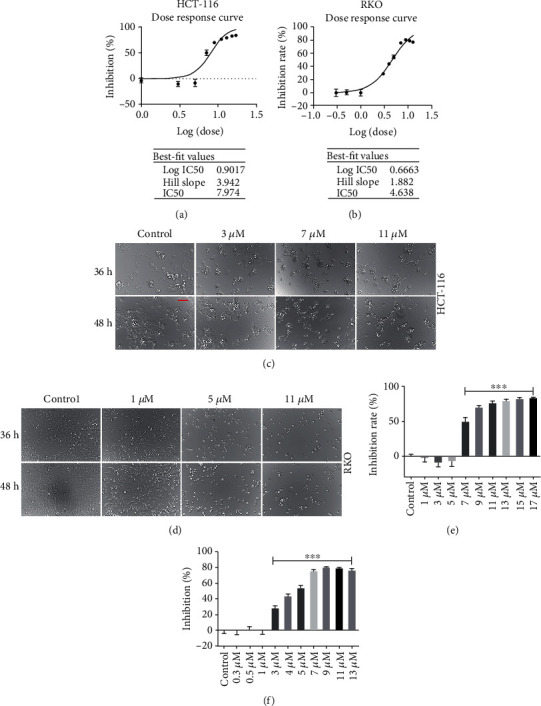
The effects of AUCAN on cell viability of HCT-116 and RKO cells. (a, b) The dose-response curve of IC50 from log(0) to log(1.5) for HCT-116 cells and from log(-1) to log(1.5) for RKO cells, *n* = 5/group. (c, d) Cell number of HCT-116 and RKO cells following AUCAN treatment in the control and AUCAN groups, respectively, *n* = 5/group. Scale bar = 50 *μ*m. (e, f) Inhibition rate of different AUCAN concentrations on HCT-116 and RKO cells. All data are shown as the mean ± SD. ^∗∗∗^*P* < 0.001. IC50: 50% inhibition concentration; h: hours.

The 50% inhibiting concentration (IC50) of RKO and HCT-116 cells was detected using CCK-8 assay to determine the sensitivity of these two cell lines in response to AUCAN treatment. Intriguingly, AUCAN proficiency showed a positive correlation with IC50 both in HCT-116 and RKO cells and inhibited cell proliferation in a dose-dependent manner. The best-fit values were 7.974 *μ*M for HCT-116 and 4.638 *μ*M for RKO cells, respectively (Figures 1(a) and 1(b)). The cell number showed a decrease after being subjected to AUCAN treatment at doses of 3 *μ*M, 7 *μ*M, and 11 *μ*M for HCT-116 cells and at doses of 1 *μ*M, 5 *μ*M, and 11 *μ*M for RKO cells at 36 h and 48 h compared to the control group (Figures 1(c) and 1(d)). Additionally, although the cellular proliferation increased gradually as the culture time prolonged, it was always suppressed with the presence of AUCAN relative to the control group (Figures 1(c) and 1(d)). The inhibition rate after AUCAN treatment in HCT-116 cells was significantly elevated at doses of 7 *μ*M, 9 *μ*M, 13 *μ*M, 15 *μ*M, and 17 *μ*M (Figure 1(e), *P* < 0.001) compared to the control group, and that in RKO cells was markedly increased at doses of 3 *μ*M, 4 *μ*M, 5 *μ*M, 7 *μ*M, 9 *μ*M, and 17 *μ*M (Figure 1(f), *P* < 0.001). These results indicated that AUCAN administration resulted in a reduction in the proliferation of HCT-116 and RKO cell lines.

### 3.2. AUCAN Suppressed Proliferation of HCT-116 and RKO Cells

**Figure 2:**
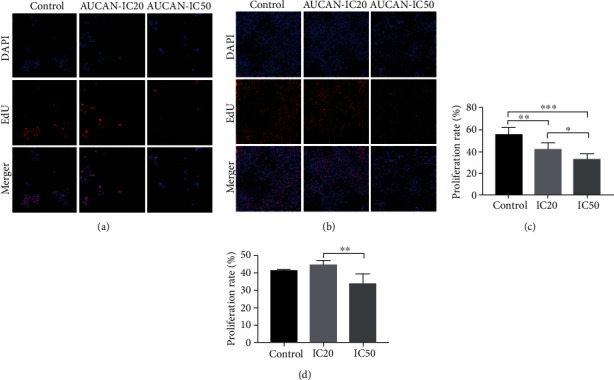
The efficacy of AUCAN treatment on the growth of HCT-116 cells and RKO cells. (a) Immunofluorescent staining of EDU for HCT-116 cells in the control, IC20, and IC50 groups. The nucleus is stained by blue, and EDU-positive cells are stained by red. (b) Immunofluorescent staining of EDU for RKO cells in the control, IC20, and IC50 groups. The nucleus is stained by blue and EDU-positive cells are stained by red. (c) The proliferation rate (EDU/DAPI) comparison of HCT-116 cells in the groups of control, IC20, and IC50. (d) The proliferation rate (EDU/DAPI) comparison of RKO cells in the groups of control, IC20, and IC50. All data are shown as the mean ± SD, *n* = 5/group. ^∗^*P* < 0.05, ^∗∗^*P* < 0.01, and ^∗∗∗^*P* < 0.001. Scale bar = 100 *μ*m. EDU: 5-ethynyl-2′-deoxyuridine; IC20: 20% inhibition concentration; IC50: 50% inhibition concentration; DAPI: 4′,6-diamidino-2-phenylindole.

To further validate the cell viability of HCT-116 and RKO cells after AUCAN administration, immunofluorescent staining of EDU was applied. As a result, the number of HCT-116 cells was significantly reduced in the IC20 group and IC50 group, especially in the IC50 group compared to the control groups (Figure 2(a)) and proliferation rate of HCT-116 cells was markedly decreased following AUCAN treatment with different doses, of which IC50 was more effective than IC20 (Figure 2(c),*P* < 0.05, *P* < 0.01, and *P* < 0.001). However, the number of RKO cells did not exhibit obvious change in the IC20 group but reduced significantly in the IC50 group compared to the control groups (Figure 2(b)), and the proliferation rate of RKO cells was markedly decreased following AUCAN IC50 treatment (Figure 2(d), *P* < 0.01). The abovementioned findings demonstrated that AUCAN could inhibit the proliferation of HCT-116 cells and higher concentration of AUCAN could suppress RKO cell proliferation.

### 3.3. AUCAN Administration Elevated the Apoptosis Rate in HCT-116 and RKO Cell Lines *In Vitro*

**Figure 3:**
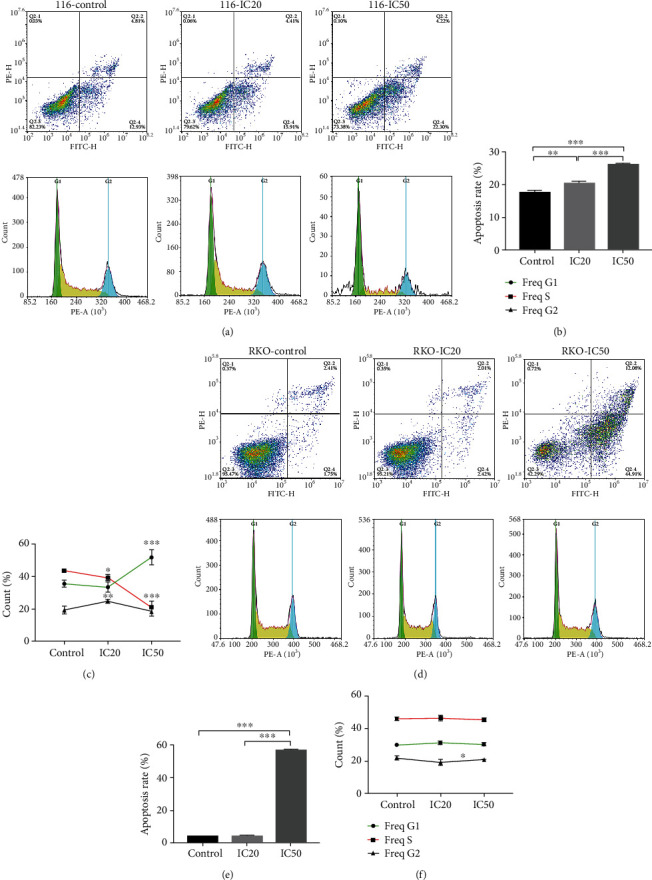
Effects of AUCAN treatment on the apoptosis of CRC cells by flow cytometry analysis. HCT-116 and RKO cells were, respectively, administrated with DMSO (control), AUCAN-IC20, and AUCAN-50 (HCT-116 cells: 8 *μ*M DMSO, 8 *μ*M AUCAN, and 3.2 *μ*M AUCAN; RKO cells: 5 *μ*M DMSO, 2 *μ*M AUCAN, and 5 *μ*M AUCAN). (a) The flow cytometry detection and (b) the apoptosis rate of HCT-116 cells in the control, IC20, and IC50 groups. (c) The cell cycle of HCT-116 cells in the control, IC20, and IC50 groups. ^∗^*P* < 0.05 vs. the control group, ^∗∗^*P* < 0.01 vs. the control group, and ^∗∗∗^*P* < 0.001 vs. the IC20 group. (d) The flow cytometry detection and (e) the apoptosis rate in the indicated groups. (f) The cell cycle of RKO cells in the control, IC20, and IC50 groups. ^∗^*P* < 0.05 vs. the IC20 group. All data are shown as the mean ± SD, *n* = 5/group. ^∗^*P* < 0.05, ^∗∗^*P* < 0.01, and ^∗∗∗^*P* < 0.001. IC20: 20% inhibition concentration; IC50: half inhibition concentration; DMSO: dimethyl sulfoxide; 116: HCT-116 cells.

Flow cytometry was also employed to detect the apoptosis rate of HCT-116 and RKO cells treated with AUCAN-IC20 and AUCAN-IC50 (Figures 3(a) and 3(d)). As shown, AUCAN-IC20 increased apoptosis rate of HCT-116 but no obvious change in RKO cells compared with the control group (Figures 3(b) and 3(e), *P* < 0.01), and both HCT-116 and RKO cells treated with AUCAN-IC50 exhibited higher apoptosis rate than that of the AUCAN-IC20 and control groups (Figures 3(b) and 3(e), *P* < 0.001). All the results revealed that AUCAN could promote the HCT-116 cell apoptosis while apoptosis promotion of RKO cells needs higher concentration. Further, in HCT-116 cells, the cell count of AUCAN-IC50 at Freq S was largely decreased, while obviously increased at Freq G1 compared with the control group (Figure 3(c), *P* < 0.001). The cell count of AUCAN-IC20 at Freq S (*P* < 0.05) and Freq G1 (*P* < 0.01) was partially decreased compared with the control group (Figure 3(c)). In RKO cells, the cell count of the AUCAN-IC50 group at Freq G2 was significantly increased compared with the AUCAN-IC20 group (Figure 3(f), *P* < 0.05); however, no obvious change was shown at other phases. These results suggested that AUCAN was involved in decreasing the survival of and enhancing apoptosis of HCT-116 cells and RKO cells, among which AUCAN-IC50 had the better effect.

### 3.4. AUCAN Treatment Decreased the Survival Rate of HCT-116 and RKO Cell Lines *In Vitro*

**Figure 4:**
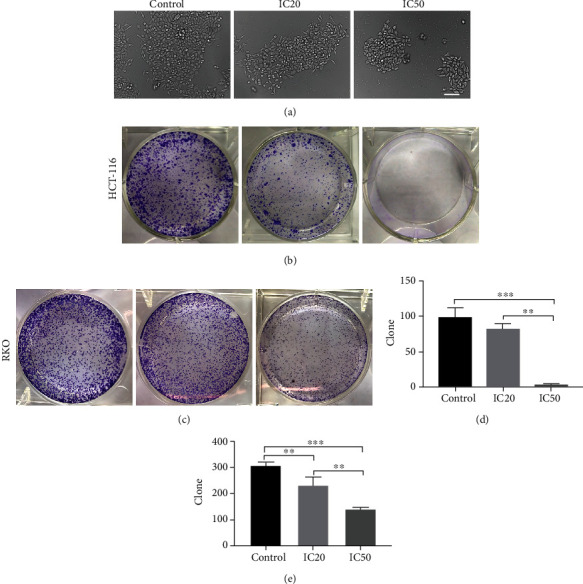
Effects of AUCAN on the survival status of CRC cells by colony formation assay. HCT-116 and RKO cells were, respectively, administrated with DMSO (control), AUCAN-IC20, and AUCAN-IC50 (HCT-116 cells: 8 *μ*M DMSO, 8 *μ*M AUCAN, and 3.2 *μ*M AUCAN; RKO cells: 5 *μ*M DMSO, 2 *μ*M AUCAN, and 5 *μ*M AUCAN). (a) The bright field images of RKO cells in the control, IC50, and IC20 groups. Scale bar = 50 *μ*m. (b, d) The colony formation assay of HCT-116 and (c, e) RKO cell lines after AUCAN administration in the control, IC50, and IC20 groups. IC20: 20% inhibition concentration; IC50: half inhibition concentration; DMSO: dimethyl sulfoxide. All data are shown as the mean ± SD, *n* = 5/group. ^∗∗^*P* < 0.01 and ^∗∗∗^*P* < 0.001.

The survival rate of HCT-116 and RKO cells was measured using the colony formation assay. RKO cells were decreased following AUCAN administration by bright field image, especially in the AUCAN-IC50 group (Figure 4(a)). Both HCT116 and RKO cells showed more cells in the control group, while less in the AUCAN-IC20 group and the least in the AUCAN-IC50 group (Figures 4(b) and 4(c)). Quantitative analysis further verified that HCT-116 and RKO cells were significantly reduced in the AUCAN-IC50 group than that in either the control (*P* < 0.001) or the AUCAN-IC20 group (Figures 4(d) and 4(e), *P* < 0.01). Also, AUCAN-IC20 decreased the RKO cell number when compared with the control group (Figures 4(d) and 4(e), *P* < 0.01). The results indicated that AUCAN could inhibit the proliferation of human-sourced colon cancer cells.

### 3.5. AUCAN Administration Lowered the Migration Rate of HCT-116 and RKO Cells *In Vitro*

**Figure 5:**
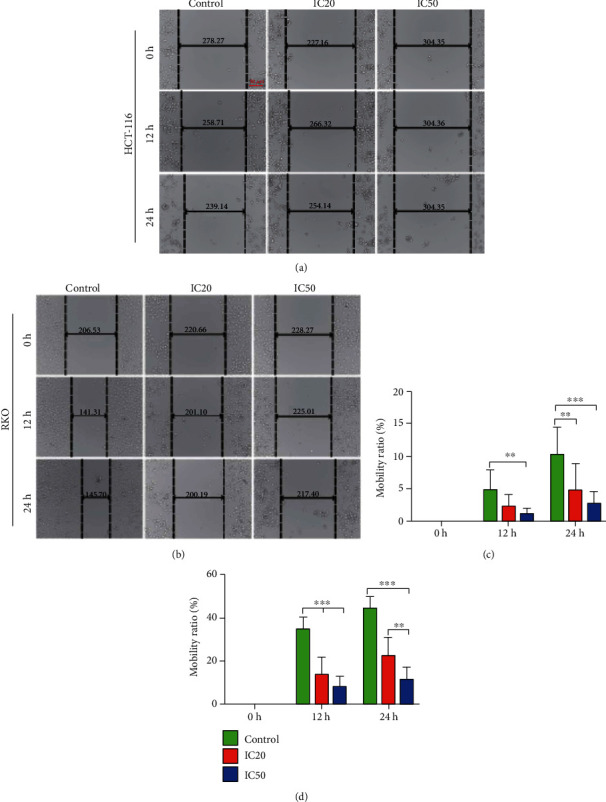
Effects of AUCAN on the metastasis of CRC cells by the wound healing test. HCT-116 and RKO cells were administered with DMSO (control) and AUCAN-IC20 and AUCAN-IC50 (HCT-116 cells: 8 *μ*M DMSO, 8 *μ*M AUCAN, and 3.2 *μ*M AUCAN; RKO cells: 5 *μ*M DMSO, 2 *μ*M AUCAN, and 5 *μ*M AUCAN). The cell migration was measured by wound healing assays in (a) HCT-116 cells and (b) RKO cells at 0 h, 12 h, and 24 h. Scale bar = 100 *μ*m. (c) The quantitative analyses of the migration rate of HCT-116 cells and (d) RKO cells. All data are shown as the mean ± SD, *n* = 5/group. ^∗∗^*P* < 0.01 and ^∗∗∗^*P* < 0.001. IC20: 20% inhibition concentration; IC50: half inhibition concentration; h: hours.

The migration rate of HCT-116 and RKO cells was tested via wound healing assays. After treated with the AUCAN for 24 h, the cell migration ability of HCT-116 and RKO was decreased (Figures 5(a) and 5(b)). For HCT-116 cells, when being cultured for 12 h, the cell migration rate was significantly decreased with AUCAN-IC50 treatment compared to that of the control group (Figure 5(c), *P* < 0.01). Additionally, 24 h later, both the administrations of AUCAN-IC50 (*P* < 0.001) and AUCAN-IC20 (*P* < 0.01) largely reduced the cell migration rate compared with that of the control group (Figure 5(c)). For RKO cells, the cell migration rate in the AUCAN-IC50 and AUCAN-IC20 groups was obviously deceased in comparison with that in the control group at 6 h, 12 h, and 24 h (Figure 5(d), *P* < 0.001). Besides, AUCAN-IC50 administration exhibited lower migration rate than AUCAN-IC20 at 24 h (Figure 5(d), *P* < 0.01). All the findings suggested that AUCAN administration could reduce the migration rate of human-sourced colon cancer cells.

### 3.6. AUCAN Treatment Decreased the Metastasis Abilities of HCT-116 and RKO Cells *In Vitro*

**Figure 6:**
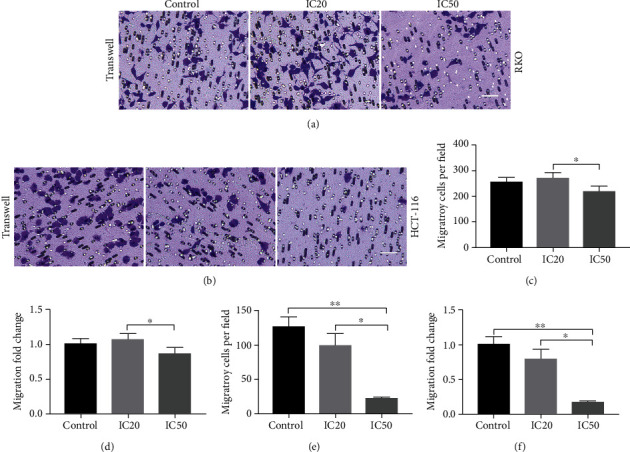
Effect of AUCAN on metastasis of HCT-116 and RKO cells *in vitro*. HCT-116 and RKO cells were, respectively, treated with DMSO (control), IC20, and IC50 of AUCAN (HCT-116 cells: 8 *μ*M DMSO, 8 *μ*M AUCAN, and 3.2 *μ*M AUCAN; RKO cells: 5 *μ*m DMSO, 2 *μ*M AUCAN, and 5 *μ*M AUCAN). The cell migration and invasion in (a) HCT-116 cells and (b) RKO cells were analyzed by Transwell assay. Scale bar = 50 *μ*m. (c, e) The migratory cells per field and (d, f) migration fold change of RKO cells and HCT-116 cells in the control, IC20, and IC50 groups. All data are shown as the mean ± SD, *n* = 5/group. ^∗^*P* < 0.05, ^∗∗^*P* < 0.01, and ^∗∗∗^*P* < 0.001. IC20: 20% inhibition concentration; IC50: half inhibition concentration.

In order to investigate the invasion rate of HCT-116 and RKO cells *in vitro*, Transwell assay was carried out. It was exhibited that the migratory cells of RKO did not reduce in the AUCAN IC20 group compared to the control group (Figure 6(a)), while AUCAN-IC50 decreased the migratory cells per field of RKO cells compared to the control group without statistical significance, but when compared with the AUCAN-IC20 group, the reduction was statistically significant (Figure 6(c), *P* < 0.05). Similar trend was revealed in the migration fold change of RKO cells (Figure 6(d), *P* < 0.05). Meanwhile, migratory cells per field of HCT-116 cells were less in the AUCAN-IC50 group than that in both the AUCAN-IC20 (*P* < 0.05) and control groups (*P* < 0.01) with corresponding smaller migration fold change in the AUCAN-IC50 group than that in the AUCAN-IC20 (*P* < 0.05) and control groups (Figures 6(e) and 6(f), *P* < 0.01).

### 3.7. AUCAN Administration Inhibited the Tumor Volume Growth in Nude Mice *In Vivo*

**Figure 7:**
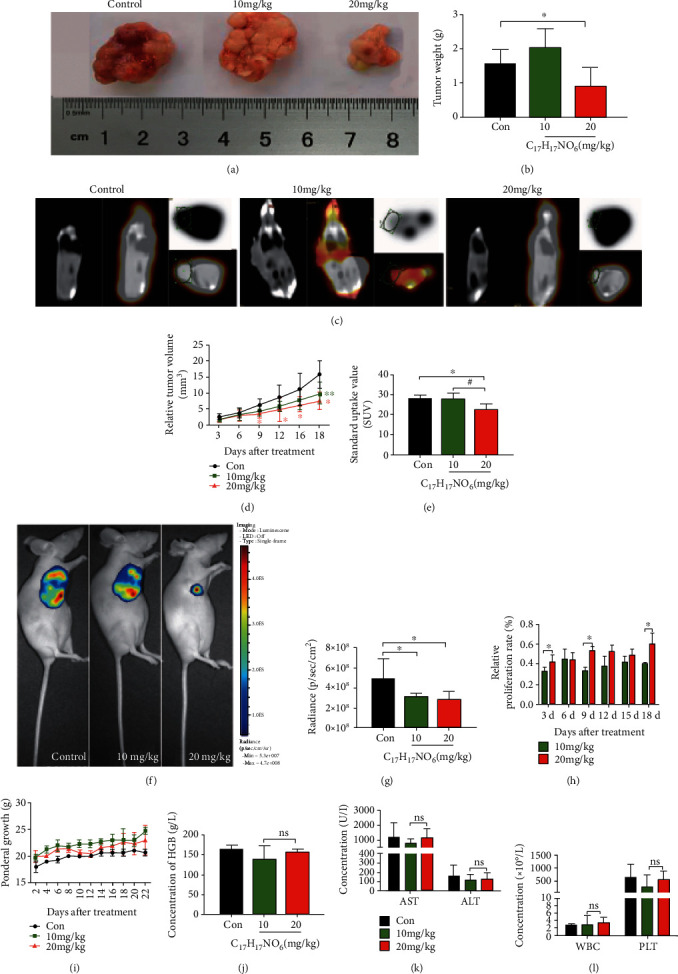
The effect of AUCAN administration on tumor growth in nude mice. The (a) morphology, (b) weight, (c) PET-CT assay, and (e) SUV of tumor in nude mice after administration of DMSO (control) and AUCAN with 10 mg/kg and 20 mg/kg. Scale bar = 1 cm. (d) The relative volume of tumor in nude mice in above groups at 3, 6, 9, 12, 15, and 18 d, respectively. (f, g) The radiance of the tumor in nude mice after the addition of DMSO (control) and AUCAN with 10 mg/kg and 20 mg/kg. (h) The proliferation rate of tumor in nude mice administered with 10 mg/kg and 20 mg/kg AUCAN at 3, 6, 9, 12, 15, and 18 d, respectively. (i) Ponderal growth of nude mice with tumor at 2, 4, 5, 8, 10, 12, 14, 16, 18, 20, and 22 d, respectively. (j) The HGB concentration of tumor and the concentration of (k) AST, ALT, (l) WBC, and PLT in the control and AUCAN with 10 mg/kg and 20 mg/kg groups. All data are shown as the mean ± SD, *n* = 10/group. ^∗^*P* < 0.05 and ^∗∗^*P* < 0.01. Con: control; C_17_H_17_NO_6_: AUCAN; d: day; HGB: hemoglobin; ns: no significance; WBC: white blood cell; PLT: platelet; AST: aspartate aminotransferase; ALT: alanine transaminase.

In order to elucidate the efficacy of AUCAN treatment on tumor growth, the RKO cells were transplanted into the nude mice. Different doses of AUCAN were tested for the therapeutic effect on tumor *in vivo*. In addition, whether there were side effects after AUCAN administration was determined through blood routine and biochemistry examination. It was demonstrated that 20 mg/kg AUCAN significantly decreased the tumor weight relative to the Control group (Figures 7(a) and 7(b), *P* < 0.05). Besides, the tumor volume was smaller after 10 mg/kg AUCAN treatment at 18 d than that of the control group (Figure 7(d), *P* < 0.01), while 20 mg/kg AUCAN obviously decreased the relative tumor volume at 9, 12, 15, and 18 d compared with the control group (Figure 7(d), *P* < 0.05). Administration of 20 mg/kg AUCAN induced a depressed glucose uptake in the tumor, demonstrated by lower SUV than that in the control and 10 mg/kg AUCAN groups (Figures 7(c) and 7(e), *P* < 0.05). Both 20 mg/kg and 10 mg/kg AUCAN administrations evidently decreased the radiance of the tumor in nude mice *in vivo* compared with the control group (Figures 7(f) and 7(g), *P* < 0.05). Meanwhile, relative to 10 mg/kg AUCAN administration at 3, 9, and 18 d, the tumor volume of nude mice was significantly increased after 20 mg/kg AUCAN administration shown by quantitative analysis of relative proliferation rate (Figure 7(h), *P* < 0.05). Nevertheless, no significant difference was exhibited in ponderal growth (Figure 7(i), *P* > 0.05). The concentration of HGB, AST, ALT, WBC, and PLT was detected to verify whether there existed side effects after AUCAN administration; however, no significant difference was revealed between 10 mg/kg and 20 mg/kg AUCAN addition in the indicated concentration tests (Figures 7(j), 7(k), and 7(l), *P* > 0.05). All the results suggested that AUCAN plays an important role in inhibiting tumor growth and has no side effects on blood routine and biochemistry examination.

### 3.8. AUCAN Administration Attenuated the Cell Proliferation and Promoted the Cell Apoptosis in Nude Mice *In Vivo*

**Figure 8:**
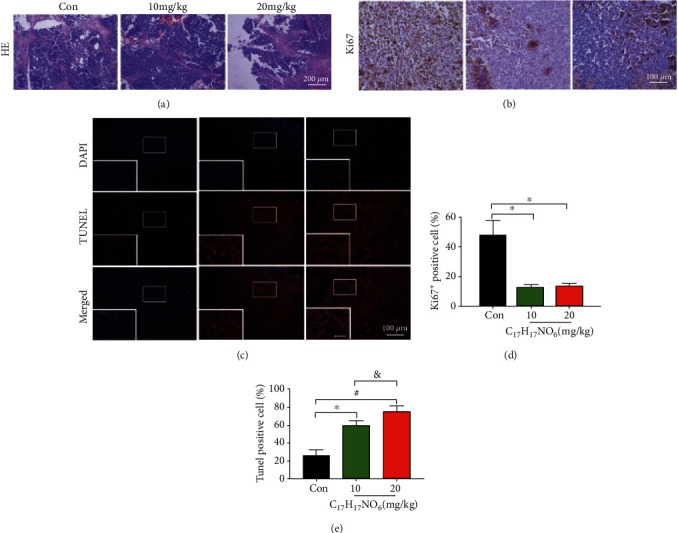
The effects of AUCAN treatment on cell proliferation and cell apoptosis in nude mice *in vivo*. (a) The HE staining of tumor tissue in nude mice *in vivo* among the control, 10 mg/kg AUCAN, and 20 mg/kg AUCAN groups. Scale bar = 200 *μ*m. (b) The immunohistochemistry Ki67 in nude mice *in vivo* among these four groups. Scale bar = 100 *μ*m. (c) The TUNEL staining of tumor tissues in nude mice *in vivo* among the indicated groups. Apoptotic cells are stained by red, and the nucleus is stained by blue. Scale bar = 100 *μ*m. (d) Ki67+ cell number and (e) TUNEL-positive cell number among the control, 10 mg/kg AUCAN, and 20 mg/kg AUCAN groups. All data are shown as the mean ± SD, *n* = 10/group. ^∗^*P* < 0.05 the AUCAN 10 mg/kg group vs. the control group, ^#^*P* < 0.05 the AUCAN 20 mg/kg vs. the control group, and ^&^*P* <0.05 the AUCAN 20 mg/kg group vs. the AUCAN 10 mg/kg group. Con: control; C_17_H_17_NO_6_: AUCAN; HE: hematoxylin and eosin staining; DAPI: 4',6-diamidino-2-phenylindole; TUNEL: terminal deoxynucleotidyl transferase dUTP-biotin nick end labeling assay.

HE staining of tumor tissue results showed the abundant RKO cell destruction in the 20 mg/kg AUCAN groups (Figure 8(a)). The immunohistochemistry of Ki67 exhibited that the positive areas of Ki67 and Ki67 cells were markedly decreased in addition of 10 mg/kg and 20 mg/kg AUCAN (Figures 8(b) and 8(d)). Additionally, TUNEL staining was employed to measure the apoptosis rate of RKO cells in nude mice *in vivo* after addition of 10 mg/kg and 20 mg/kg AUCAN (Figure 8(c)), showing that both 10 mg/kg and 20 mg/kg AUCAN additions evidently elevated the number of apoptotic RKO cells, while 20 mg/kg AUCAN addition was more effective (Figure 8(e), *P* < 0.05), suggesting that AUCAN plays an important role in promoting colorectal tumor cell apoptosis in nude mice *in vivo*.

### 3.9. Screening of Differentially Expressed Proteins (DEPs) in AUCAN-Administered RKO Cells

**Figure 9:**
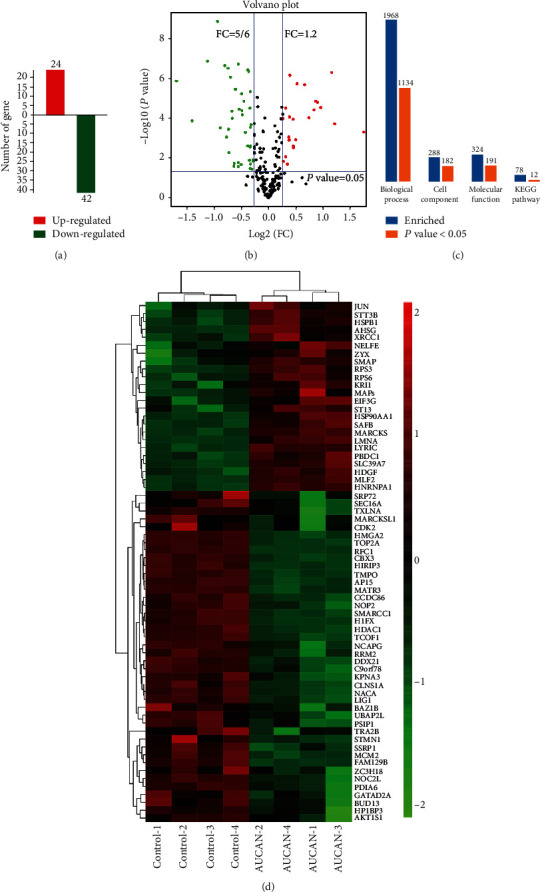
Screening of DEPs. (a) Identification of up- and downregulated proteins. (b) DEPs were exhibited by volcano plot. Fold change > 1.2 or <5/6 and *P* < 0.05 is considered to be significantly differentially expressed. Red indicates upregulated proteins, green for downregulated ones, and black for proteins without differential expression. (c) Enrichment analysis of biological functions of DEPs. (d) Heat maps of identified proteins in the control and AUCAN groups. All data are shown as the mean ± SD, *n* = 4/group. DEPs: differentially expressed proteins.

We detected the differences among the samples collected before in the control and AUCAN groups, of which 24 were upregulated and 42 were downregulated (Figures 9(a) and 9(d)). DEPs were concealed via volcano plot, red for upregulated proteins, green for downregulated ones, and black for no DEPs (Figure 9(b)). In order to understand the relationships and discrepancy of samples more intuitively and comprehensively, we conducted enrichment analysis using the union set of the DEPs identified above. Of 1968 enriched biological processes, 1134 were of statistical significance; of 268 enriched cell components, 182 were of statistical significance; of 324 enriched molecular functions, 191 were of statistical significance; of 78 enriched KEGG pathways, 12 were of statistical significance (Figure 9(c)).

### 3.10. Gene Ontology (GO) and Kyoto Encyclopedia of Genes and Genomes (KEGG) Pathway Analysis of DEPs in AUCAN-Administered RKO Cells

**Figure 10:**
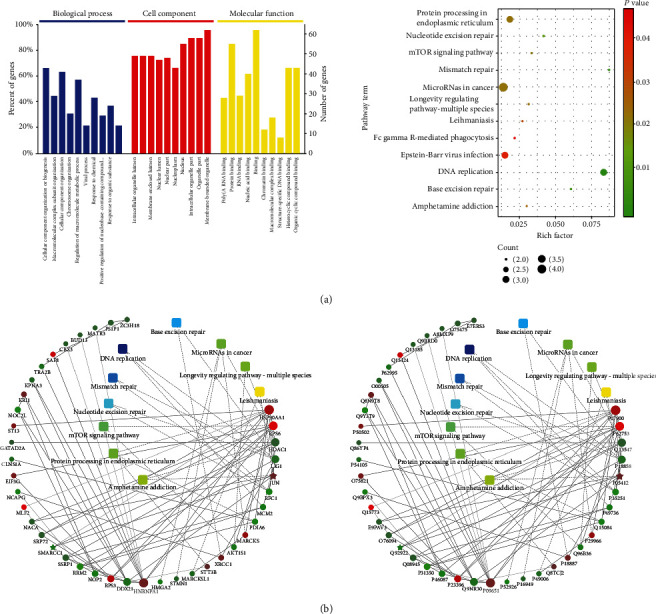
GO and pathway enrichment analysis of DEPs. (a, b) GO analysis and KEGG analysis of DEPs. (b) Protein-protein interaction networks of DEPs. Rich factor represents the ratio of DEP number annotated in this pathway term to all protein number annotated in this pathway term. Red dot indicates upregulated protein, and green for downregulated one. Biological processes, cellular localization, molecular functions, or signaling pathways were presented by rectangles. Blue indicates higher *P* value while yellow for lower. Solid lines represent interrelated protein (genes)-proteins (genes), and dashed lines represent interrelated metabolic pathways-proteins (genes). All data are shown as the mean ± SD. GO: Gene Ontology; KEGG: Kyoto Encyclopedia of Genes and Genomes pathway analysis.

Functional annotation of the above-identified proteins was performed in accordance with the annotation information from GO database and KEGG database. The same types of proteins were gathered in a cluster with similar biological functions. According to the enrichment factor, the top 10 biological processes were cellular component organization or biogenesis, macromolecular complex subunit organization, cellular component organization, chromosome organization, regulation of macromolecule metabolic process, viral process, response to chemical, positive regulation of nucleobase-containing compound, response to organic substance, and symbiosis encompassing mutualism through parasitism. As for cell component, the top 10 were selected: intracellular organelle lumen, organelle lumen, membrane-enclosed lumen, nucleus lumen, nuclear part, nucleoplasm, nucleus, intracellular organelle part, organelle part, and membrane-bounded organelle. The top 10 molecular functions were poly(A) RNA binding, protein binding, RNA binding, nuclei acid binding, binding, chromatin binding, macromolecular complex binding, structure-specific DNA binding, heterocyclic compound binding, and organic cyclic compound binding (Figure 10(a)). Pathway enrichment analysis of the DEPs was also conducted based on the KEGG database in order to explore the changes of metabolic pathways. Twelve pathways included protein processing in the endoplasmic reticulum, nucleotide excision repair, mTOR signaling pathway, mismatch repair, microRNAs in cancer, longevity regulating pathway-multiple species, leishmaniasis, Fc gamma R-mediated phagocytosis, Epstein-Barr virus infection, DNA replication, base excision repair, and amphetamine addition were significantly enriched (Figure 10(b)). Interestingly, HSP90AA1 showed close interactions with upregulated genes like ST13, MLF2, and RPS3, and DNA replication signaling pathway exhibited relation with downregulated genes like LIG1, RFC1, and MCM2, which provide referential significance for future investigations.

## 4. Discussion

In this study, we firstly identified the ability of AUCAN to inhibit the proliferation, invasion, and migration, as well as induce the apoptosis of tumor cells by applying HCT-116 and RKO cells. Moreover, AUCAN application in nude mice demonstrated its effects on alleviating CRC, including decreasing the tumor weight and volume. An in-depth investigation of the underlying mechanisms was carried out using proteomic analysis, which proved AUCAN played an important role in treating CRC.

AUCAN, a newly invented anticancer agent (patent ID: 201710388136.8), has been applied in lung cancer, liver cancer, bladder cancer, breast cancer, and nasopharyngeal carcinoma *in vitro*, in which cellular metabolism, proliferation, and cell cycle distribution were obviously affected [10]. It was reported AUCAN could efficiently depress cell proliferation and induce cell apoptosis of glioma cells, and its combination with AUCAN was elucidated to have a more promoting effect [10]. In our present study, with the increasing dose of AUCAN, the inhibition rate of tumor cells was elevated gradually. AUCAN-IC20 and AUCAN-IC50 applied in the later experiment obviously revealed that the proliferation rate and migration rate both in HCT-116 and RKO cells were much lower with AUCAN-IC50 administration than the AUCAN-IC20 and control groups. Additionally, AUCAN-IC50 markedly decreased the growth and volume of CRC tumor in nude mice shown by PET-CT and *in vivo* morphological experiments. Importantly, AUCAN exerts no significant adverse effect indicated by normal weight gain in mice and no significant changes in blood serum biochemical indices. For the first time, the present study identified the efficacy of novel anticancer drug for impeding tumor growth and migration *in vitro* and *in vivo*. Proteins function as critical regulators of biological processes and link to the risk of diseases and clinical outcomes [13, 14]. In the past 20 years, proteomics has constantly developed and matured, exerting transformative influence on varieties of diseases [15]. With technology enhancement, hundreds and thousands of proteins can be instantly measured and analyzed [16]. In this study, DEPs in the antitumor activities of AUCAN were identified by phosphorylated proteomics analysis, and twenty-four upregulated proteins as well as forty-two downregulated proteins were shown, implying the AUCAN treatment in colorectal cancer involves a multiple-gene regulated process. The functional clustering analysis revealed that the abovementioned DEPs took participation in various biological processes, cellular component, molecular function, and numerous correlated signal pathways. Initially, biological function analysis elucidated that the identified proteins were mainly involved in cellular component organization or biogenesis, macromolecular complex subunit organization, cellular component organization, chromosome organization, regulation of macromolecule metabolic process, viral process, response to chemical, positive regulation of nucleobase-containing compound, response to organic substance, and symbiosis encompassing mutualism through parasitism. Besides, the cellular analysis revealed the DEPs were mainly contained in the intracellular organelle lumen, organelle lumen, membrane-enclosed lumen, nucleus lumen, nuclear part, nucleoplasm, nucleus, intracellular organelle part, organelle part, and membrane-bounded organelle. Moreover, molecular function analysis uncovered that the DEPs mainly participated in poly(A) RNA binding, protein binding, RNA binding, nucleic acid binding, binding, chromatin binding, macromolecular complex binding, structure-specific DNA binding, heterocyclic compound binding, and organic cyclic compound binding. All these findings provided evidence for the conclusion in the previous study stating that the anticancer effect is achieved by affecting cell metabolism, proliferation, and cell cycle distribution [10]. Moreover, pathway enrichment analysis of the DEPs explored the changes of metabolic pathways. The top 12 pathways were as follows: protein processing in the endoplasmic reticulum, nucleotide excision repair, mTOR signaling pathway, mismatch repair, microRNAs in cancer, longevity regulating pathway-multiple species, leishmaniasis, Fc gamma R-mediated phagocytosis, Epstein-Barr virus infection, DNA replication, base excision repair, and amphetamine addition. Interestingly, DNA replication pathway showed interactions with downregulated genes like LIG1, RFC1, and MCM2, and mismatch repair exhibited relation with downregulated gene—P18858 and P35251. LIG1, an encoded protein, functions in DNA replication, recombination, and base excision repair process, whose disruption may be associated with varieties of cancers [17]. RFC1 is a DNA-dependent ATPase essential for eukaryotic DNA replication and repair and it has a crucial role in telomere stability [18].

## 5. Conclusion

Summarily, our findings displayed the anticancer efficacy of AUCAN in colorectal cancer. A novel therapeutic agent for colorectal cancer may be potential for clinical colorectal cancer treatment, and our outcome may provide data resource based on the proteomic analysis.

## Data Availability

The data used to support the findings of this study are included within the article.

## Abbreviations

CRC: Colorectal cancer
TCM: Traditional Chinese medicine
HCT-116: HCT-116 cell lines
RKO: RKO colorectal carcinoma cell lines
SPF: Specific pathogen-free
IC20: 20% inhibition concentration
IC50: 50% inhibition concentration
EDU: 5-Ethynyl-2′-deoxyuridine
TUNEL: Terminal deoxynucleotidyl transferase-mediated nick end labeling
DAPI: 4′,6-Diamidino-2-phenylindole
DMSO: Dimethyl sulfoxide
HE: Hematoxylin and eosin staining
GO: Gene Ontology
KEGG: Kyoto Encyclopedia of Genes and Genomes pathway analysis
HGB: Hemoglobin
AST: Aspartate aminotransferase
ALT: Alanine transaminase
WBC: White blood cell
PLT: Platelet
d: Days
h: Hours
min: Minutes.
